# Prevalence, Serotype, Antibiotic Susceptibility, and Genotype of *Salmonella* in Eggs From Poultry Farms and Marketplaces in Yangling, Shaanxi Province, China

**DOI:** 10.3389/fmicb.2020.01482

**Published:** 2020-08-04

**Authors:** Wei Li, Hao Li, Shujuan Zheng, Zewei Wang, Huanjing Sheng, Chunlei Shi, Xianming Shi, Qinya Niu, Baowei Yang

**Affiliations:** ^1^College of Food Science and Engineering, Northwest A&F University, Yangling, China; ^2^MOST-USDA Joint Research Center for Food Safety, School of Agriculture and Biology, Shanghai Jiao Tong University, Shanghai, China

**Keywords:** antibiotic resistance, eggs, genotype, *Salmonella*, serotype

## Abstract

Poultry products such as eggs provide essential nutrients to the human body and thus play vital roles in the human food network. *Salmonella* is one of the most notorious foodborne pathogens and has been found to be prevalent in eggs. To better understand the characteristics of *Salmonella* in eggs, we investigated the prevalence of *Salmonella* spp. in 814 fresh eggs collected from poultry farms and retail marketplaces in Yangling, Shaanxi Province, China. The serotype, genotype, and antibiotic susceptibilities of 61 *Salmonella* isolates recovered from the eggs were analyzed. The average detection rate of *Salmonella*-positive eggs was 5.6%, with 6.6% of the eggs collected from poultry farms and 5.1% from marketplaces. Thirteen serotypes were identified from the 61 isolates, among which *Salmonella* Typhimurium (24.5%) and *Salmonella* Indiana (22.9%) were the most prevalent serotypes. Other dominant serotypes included *Salmonella* Thompson (13.1%) and *Salmonella* Enteritidis (11.4%), with the remaining nine serotypes detected at low rates (1.6–4.9%). All the *Salmonella* isolates tested were resistant to sulfisoxazole (100.0%). The majority (77.1%) of the isolates were resistant to nalidixic acid, amoxicillin-clavulanate, and ampicillin, while nearly two-thirds (63.9–68.9%) were resistant to trimethoprim–sulfamethoxazole, kanamycin, tetracyclines, and chloramphenicol. The rate of resistance to ciprofloxacin was 40.1%; the resistance rates to streptomycin, ceftiofur, and ceftriaxone ranged from 21.3 to 26.2%; and those to gentamicin, amikacin, and cefoxitin were relatively low (3.3–16.4%). Forty-nine (80.3%) *Salmonella* isolates exhibited resistance to multiple antibiotics, 20 (32.8%) of which were resistant to at least 10 antibiotics. Subtyping by pulse-field gel electrophoresis revealed a close genetic relatedness of *Salmonella* isolates from poultry farms, in striking contrast to the high diversity of the isolates obtained from marketplaces. Isolates of the same serotype always shared identical genotype and antibiotic resistance profiles, even the ones that were recovered from eggs sampled at different locations and times. These findings indicate that diverse *Salmonella* spp. with high rates of multidrug resistance are prevalent in fresh eggs in the study area. More attention should be paid to egg production, transportation, and storage to prevent foodborne outbreaks caused by *Salmonella*.

## Introduction

*Salmonella* spp. are notorious foodborne pathogens that can cause diarrhea in humans and animals (Cowen et al., 2016). Reportedly, there are approximately 9.38 million cases of *Salmonella* infections and 15,000 deaths from the infection worldwide every year (Patchanee et al., 2017). Among the various vehicles of *Salmonella*, poultry and poultry products are not only identified as the predominant reservoirs, but are also considered to be the primary sources of human salmonellosis based on evidence from epidemiological traceback investigations (Andino and Hanning, 2015; McWhorter and Chousalkar, 2019). A previous study has indicated that the egg white possesses unique physical and biochemical properties, creating a complex antimicrobial environment to resist antigens (Huang et al., 2019). However, egg white and egg yolk membranes are the major infection sites for *Salmonella*, which means that *Salmonella* still has the opportunity to survive in the resistant environment of eggs (Raspoet et al., 2019).

Currently, the outbreak of foodborne diseases caused by the consumption of eggs contaminated with *Salmonella* remains severe worldwide. According to reports from the United States Food and Drug Administration (FDA) and the Centers for Disease Control and Prevention (CDC), there were 52 foodborne outbreaks in Missouri in 2015 due to eggs contaminated by *Salmonella* Oranienburg. In 2016, the CDC again notified the FDA of eight clinical cases from three states, i.e., Illinois, Kansas, and Missouri, which were closely related to the hereditary strains of the *S.* Oranienburg outbreak in 2015 (FDA, 2019c). In March 2018, the FDA learned about 45 *Salmonella* Braenderup-infected consumers in 10 states, 11 of whom were hospitalized without death. The outbreak was tracked down to Rose Acre Farms’ Hyde County farm in North Carolina and 207 million eggs were recalled around the United States (FDA, 2019a).

Recently, *Salmonella* was detected in eggs collected from 41 (63.5%) of 63 farms in Australia that underwent environmental sampling (Moffatt et al., 2017). In addition, 88.6% (124/140) of the eggs and all of the 19 farms sampled in the western region of Cameroon were found to be *Salmonella*-positive (Kouam et al., 2018). So far, multiple *Salmonella* serotypes including *Salmonella* Typhimurium, *Salmonella* Indiana, and *Salmonella* Enteritidis have been commonly detected from eggs, poultry, and poultry farm environments (Moffatt et al., 2017). To prevent infection by pathogenic bacteria and promote the growth of laying hens, a large number of antibiotics are used in the feeding process. A nationwide market survey indicated that the total amount of 36 antibiotics that are frequently detected in livestock farms, wastewater treatment plants, and environment settings reached 92,700 tons in China in 2015. Notably, the rate of antibiotic usage in poultry farms was as high as 19.6% (18,100/92,700) (Zhang et al., 2015).

β-Lactams, cephalosporins, and fluoroquinolones are the most frequently used antibiotics in the poultry industry (Fardsanei et al., 2017). Taking into account the antibiotics used in other fields, *Salmonella* has developed high resistance to a broad range of antibiotics, leading to increased healthcare costs and clinical treatment failure (Cui et al., 2016). Many studies have characterized *Salmonella* in poultry and poultry-associated foods globally, especially eggs (Moffatt et al., 2017; Kouam et al., 2018; Li et al., 2018; FDA, 2019a,b,c; Karimiazar et al., 2019; Kingsbury et al., 2019; Sanchez-Salazar et al., 2019). Evidence suggests that the genetic diversity of *Salmonella* isolated from various sources is abundant, whereas the isolates from the same poultry farms are closely related (Xie et al., 2019). Moreover, the occurrence and characteristics of *Salmonella* in poultry products including eggs always show dynamic variation during production, storage, and handling (Li et al., 2018). Therefore, it is of great significance to continuously monitor this group of pathogens in eggs to ensure food safety.

In this study, we characterized *Salmonella* isolates from fresh eggs in poultry farms and retail marketplaces in Yangling, Shaanxi Province, China. The objective of the study was to further explore the prevalence and transmission of *Salmonella* during egg production and sales links in order to prevent *Salmonella* outbreaks.

## Materials and Methods

### Sample Collection

A total of 814 fresh eggs were collected in Yangling and its surrounding districts in Shaanxi Province, China, from mid-2013 to late 2014. Specifically, 304 eggs were collected from three different poultry farms. Farm X in Xiajiagou Village is a large modern laying hen farm with fully automated cage raising equipment, including equipment for heating, ventilation, water supply, feeding, egg collection, manure removal, cages, and lighting. In this farm, there are approximately 20,000 laying hens with a breeding density of 9–10/m^2^. Farm C in Cuixigou Village is a semi-automated laying hen farm of a medium production scale, with basic equipment for heating, ventilation, cages, lighting, and water feeding. In this farm, there are approximately 9000 laying hens with a breeding density of 11–12/m^2^. Farm F in Fuzeyuan Company is the smallest laying hen farm with basic equipment for heating, ventilation, cages, and lighting. In this farm, there are approximately 4000 laying hens with a breeding density of 9–10/m^2^. Additionally, 510 eggs of seven brands were collected from seven supermarkets (eight retail outlets) and four wet markets (10 retail outlets).

At each sampling location, the eggs were sampled weekly in August, September, and October 2013 and in March, June, August, October, and November 2014. At each sampling time, three, six, or nine eggs (depending on the total number of eggs for sale) were collected at random in a supermarket or wet market. In each poultry farm, no more than 50 eggs were collected at random from egg-laying troughs. The egg samples were delivered to the Microbiology Laboratory in the College of Food Science and Engineering, Northwest A&F University (Yangling, Shaanxi Province China) for *Salmonella* isolation within 12 h of collection.

### Isolation and Identification of *Salmonella*

*Salmonella* isolates were recovered from egg samples according to the method described by Yang et al. (2010) with some modifications. Briefly, each egg (ca. 60 g, including the shell) was broken and uniformly mixed with 540 mL of buffered peptone water (BD, Cockeysville, MD, United States) in a homogenization bag, then homogenized for 2 min. Before handling the next egg, a new pair of germ-free gloves was used to avoid bacterial cross-contamination throughout the process. The mixed cultures were pre-incubated at 37°C with shaking at 100 rpm for 6 h. Subsequently, 10 mL cultures were transferred into 100 mL of tetrathionate broth (TT; BD) and 1 mL cultures were transferred to 100 mL of Rappaport–Vassiliadis broth (RV; BD). The inoculated TT and RV broths were incubated at 42°C with shaking at 100 rpm for 24 h. Afterward, TT cultures were streaked on to xylose lysine tergitol 4 agar (XLT4; BD) and RV cultures were streaked on to xylose lysine deoxycholate agar (XLD; BD). The inoculated XLT4 and XLD plates were incubated at 35°C for 48 h, and two putative colonies with a typical *Salmonella* phenotype were picked from each plate and subcultured on a fresh XLT4 plate for purification. The purified isolates were stabbed into one triple sugar iron (BD) slant and one urea-agar (BD) slant, respectively, followed by incubation at 35°C for 18–24 h to exclude *Escherichia coli* and *Proteus* species.

Presumptive *Salmonella* isolates were identified by polymerase chain reaction (PCR) using the primers *inv*AF (5′-GTGAAATTATCGCCACGTTCGGGCAA-3′) and *inv*AR (5′-TCATCGCACCGTCAAAGGAACC-3′) (Rahn et al., 1992). Polymerase chain reaction was carried out in a 25-μL reaction mixture containing 0.5 μM of each primer, 250 μM of dNTP, 1 × PCR buffer, 1.5 mM MgCl_2_, 0.5 U of *Taq* DNA polymerase (TaKaRa, Dalian, China), and 5 μL of DNA template. All PCR reactions were performed on a MyCircle thermocycler (Bio-Rad, Hercules, CA, United States) with pre-denaturation at 95°C for 5 min, followed by 35 cycles of 95°C for 30 s, 64°C for 30 s, and 72°C for 30 s, and a final extension of 72°C for 5 min. Polymerase chain reaction products were stained with ethidium bromide and visualized under UV light after gel electrophoresis in 1% agarose (Kasturi and Drgon, 2017). *S.* Typhimurium LT2 was used as the positive control strain.

### Serotyping of Somatic and Flagellar Antigens

After PCR identification, *Salmonella* isolates were serotyped in the Henan Center of Disease Control and Prevention (Zhengzhou, Henan Province, China). Somatic (O) and flagellar (H) antigens were determined by the slide agglutination method using *Salmonella*-specific hyperimmune sera (S&A Company, Bangkok, Thailand). The serotype was identified by the antigen form according to the Kauffman–White scheme (Ben Aissa et al., 2007). *S.* Typhimurium LT2 was used as the positive control strain.

### Antibiotic Susceptibility Test

Table 1 lists the antibiotics used for the susceptibility test. The agar dilution method suggested by the Clinical and Laboratory Standards Institute (CLSI, 2015) was used to determine the minimum inhibitory concentrations of the antibiotics to *Salmonella* isolates on Mueller-Hinton agar (Beijing Land Bridge Technology Co., Ltd, Beijing, China). The results of resistance, intermediate resistance, and susceptibility were interpreted via the guidelines established by the CLSI, except for streptomycin, the breakpoint of which was interpreted by that specified by the National Antimicrobial Resistance Monitoring System (U.S. Food and Drug Administration, 2013; FAO, 2018a,b). *E. coli* ATCC25922 and ATCC35218 and *Enterococcus faecalis* ATCC29212 were used as the quality control strains.

**TABLE 1:** Antibiotic concentration ranges and data interpretation for the susceptibility test of *Salmonella* isolates.

**Antibiotic agent**	**Abbreviation**	**Antibiotic concentration range (μg/mL)**	Breakpoint interpretive criteria (μg/mL)*		
			**S**	**I**	**R**
Aminoglycosides					
Amikacin	AMK	0.5–64	≤16	32	≥64
Gentamicin	GEN	0.25–16	≤4	8	≥16
Kanamycin	KAN	1–64	≤16	32	≥64
Streptomycin ^#^	STR	4–64	≤32	NA	≥64
Penicillin β-lactamase inhibitor combinations					
Amoxicillin–clavulanic acid	AMC	0.25/0.12–32/16	≤8/4	16/8	≥32/16
Ampicillin	AMP	0.5–32	≤8	16	≥32
Cephalosporins (3rd and 4th generations)					
Cefoxitin	FOX	2–32	≤8	16	≥32
Ceftriaxone	CRO	0.03–4	≤8	16-32	≥64
Ceftiofur	TIO	0.25–8	≤8	16-32	≥64
Quinolone					
Nalidixic acid	NAL	1–32	≤16	NA	≥32
Fluoroquinolones					
Ciprofloxacin	CIP	0.004–4	≤0.06	0.12–0.5	1≥
Tetracyclines					
Tetracyclines	TET	0.5–16	≤4	8	≥16
Chloramphenicol					
Chloramphenicol	CHL	2–32	≤8	16	≥32
Folate pathway inhibitors					
Sulfisoxazole	FIS	8–512	≤256	NA	≥512
Trimethoprim-sulfamethoxazole	SXT	0.5/9.5-4/76	≤2/38	NA	≥4/76

*S, sensitive; I, intermediate resistance; and R, resistance. ^#^No CLSI interpretative criteria are available for streptomycin; thus, provisional breakpoint from NARMS was used.

### Subtyping by Pulse-Field Gel Electrophoresis

Pulse-field gel electrophoresis (PFGE) was used to determine the genetic relationship between *Salmonella* isolates according to the PulseNet protocol (Centers for Disease Control and Prevention [CDC], 2013). Briefly, *Salmonella* subcultures were grown on Luria–Bertani agar overnight at 37°C to reach an optical density of 0.5 and then embedded in SeaKem Gold agarose (Lonza, Rockland, ME, United States). Then culture plugs were lysed in cell lysis buffer with 100 μg mL^–1^ protease K (TaKaRa) by incubation at 55°C in a shaking water bath for 2 h. Subsequently, the lysed plugs were washed twice with sterile water and then washed four times with sterilized Tris–EDTA buffer. A slice was cut from the plug and digested using 50 U of *Xba*I (TaKaRa) by incubation in a 37°C water bath for 1.5–2 h. The digested DNA fragments in each slice were separated in 0.5 × Tris-borate-EDTA buffer at 14°C for 20 h using a Chef Mapper electrophoresis system (Bio-Rad) with pulse times of 2.16–63.8 s. The gel was stained using ethidium bromide (100 μg mL^–1^), and the DNA bands were visualized via UV transillumination (Bio-Rad). Pulse-field gel electrophoresis results were manually analyzed using BioNumerics v3.0 (Applied Maths, Kortrijk, Belgium), and the extent of variability was determined using the Dice coefficient and clustering by the unweighted pair-group average method (Yang et al., 2014). *S.* Braenderup H9812 was used as the standard DNA control.

### Statistical Analysis

Statistical analysis was performed using IBM SPSS Statistics v22 (IBM Corp., Armonk, NY, United States). Pearson’s chi-square test was used to determine the differences in the detection rates of *Salmonella*-positive samples and *Salmonella* serotypes in eggs across different sampling locations (i.e., wet markets, supermarkets, and poultry farms) and times. The results were analyzed at the 5% (α = 0.05) level to determine whether differences were significant. Data were edited using Microsoft Excel 2016 (Microsoft Corp., Redmond, WA, United States), with environmental variables (i.e., serotype, sampling location, and sampling time) and explanatory environmental variables (i.e., number and category of the antibiotics to which *Salmonella* exhibited resistance) organized into the form of “row representing samples and column representing variables.” The edited data were imported into Canoco v5.0 (Microcomputer Power, Ithaca, NY, United States) for redundancy analysis. The rate of antibiotic-resistant *Salmonella* isolates between different serotypes was analyzed using R v3.4.4^1^, and a heatmap was created using the R package “pheatmap” v3.0.1^2^.

## Results

### Prevalence of *Salmonella*

Of the 814 eggs, 46 (5.6%) were detected to be *Salmonella*-positive (Table 2). The detection rate of *Salmonella*-positive eggs from poultry farms was slightly higher (6.6%, 20/304) than that from retail markets (5.1%, 26/510). Among the different marketplaces, the detection rate of *Salmonella*-positive eggs was slightly higher in supermarkets (5.8%, 15/259) than in wet markets (4.4%, 11/251). However, the differences in the detection rates of *Salmonella*-positive eggs were not significant among retail markets and poultry farms.

**TABLE 2:** Prevalence of *Salmonella* in eggs collected from poultry farms and marketplaces at different sampling locations and times.

	**Location or time (egg number)**	**Number (percentage) of *Salmonella*-positive eggs**	**Number (percentage) of *Salmonella* isolates**
Source	Retail market (510)	26 (5.1)	40 (65.6)
	Poultry farm (304)	20 (6.6)	21 (34.4)
Retail	Supermarket (259)	15 (5.8)	22 (36.1)
market	Wet market (251)	11 (4.4)	18 (29.5)
Poultry	C (88)	19 (21.6)^##^	20 (32.8)
farm	X (108)	1 (0.9)	1 (1.6)
	F (108)	0	0
Sampling	2013 (134)	2 (1.5)	2 (3.3)
year	2014 (680)	44 (6.5)*	59 (96.7)
Sampling	Aug. 2013 (98)	2 (2.0)^c^	2
month	Spt. 2013 (12)	0^c^	0
	Oct. 2013 (24)	0^c^	0
	Mar. 2014 (66)	20 (30.3)^a^	33
	Jun. 2014 (175)	4 (2.3)^c^	5
	Aug. 2014 (135)	0^c^	0
	Oct. 2014 (196)	19 (9.7)^b^	20
	Nov. 2014 (108)	1 (0.9)^c^	1
Total	814	46 (5.6)	61 (100)

^##^*P* < 0.01; *P < 0.05, *P* = 0.023. ^*a,b,c*^Values (percentage) with different superscripted letters are significantly different between groups.

Among the three poultry farms, no *Salmonella*-positive eggs were detected from farm F, only one (0.9%) *Salmonella*-positive egg was detected in farm X, while 19 (21.6%) were detected in farm C. The detection rate of *Salmonella* in eggs from farm C was significantly (*P* < 0.01) higher than those from the other two poultry farms. During the investigation period, the detection rate of *Salmonella*-positive eggs was significantly higher (*P* < 0.05) in 2014 (6.5%, 44/680) than in 2013 (1.5%, 2/134). Additionally, the detection rates of *Salmonella-*positive eggs in March 2014 (30.3%, 20/60) and October 2014 (9.7%, 19/196) were significantly (*P* < 0.05) higher than those from the other sampling times (Table 2).

In total, 61 *Salmonella* isolates were recovered from XLD and XLT4 plates derived from the 46 *Salmonella*-positive eggs. Among them, 40 (65.6%) isolates were obtained from eggs collected from retail markets and 21 (34.4%) were from eggs sampled from poultry farms (Table 2).

### Serotype of *Salmonella*

**FIGURE 1:**
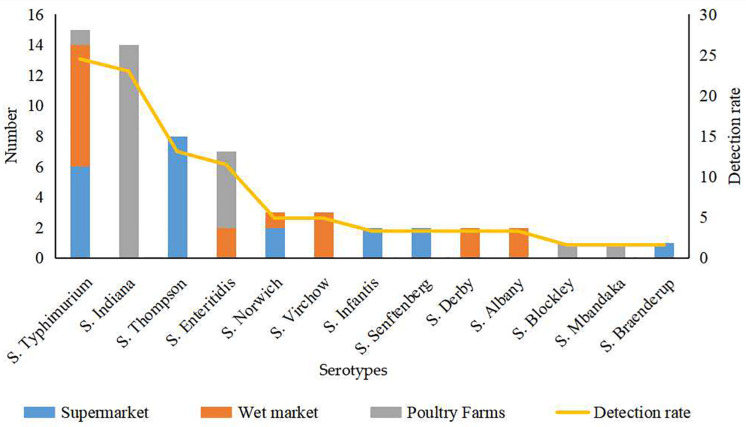
Serotype of 61 *Salmonella* isolates from eggs from different sources.

Thirteen serotypes were identified from the 61 *Salmonella* isolates, among which *S.* Typhimurium (24.5%) and *S.* Indiana (22.9%) were the most commonly detected serotypes (Figure 1). The detection rates of *Salmonella* Thompson (13.1%) and *Salmonella* Enteritidis (11.4%) were moderately high. Additionally, *Salmonella* Norwich and *Salmonella* Virchow accounted for 4.9% of all the identified isolates each; *Salmonella* Derby, *Salmonella* Senftenberg, *Salmonella* Infantis, and *Salmonella* Albany accounted for 3.2% each; and *Salmonella* Blockley, *Salmonella* Mbandaka, and *S.* Braenderup were detected at the lowest rates (1.6% each).

The 39 *Salmonella* isolates recovered from retail eggs covered 10 serotypes, while the 22 isolates recovered from poultry farms covered five serotypes (Figure 1). Considering their source, all *S.* Indiana, *S.* Blockley, and *S.* Mbandaka isolates and the majority (71.4%) of *S.* Enteritidis isolates were from poultry farms. All *S.* Thompson, *S.* Infantis, *S.* Senftenberg, and *S.* Braenderup isolates and 40.0% of *S.* Typhimurium isolates were from supermarkets. All *S.* Virchow, *S.* Derby, and *S.* Albany isolates and more than half of *S.* Norwich (66.7.%) and *S.* Typhimurium (53.3%) isolates were from wet markets.

### Antibiotic Susceptibility of *Salmonella*

**FIGURE 2:**
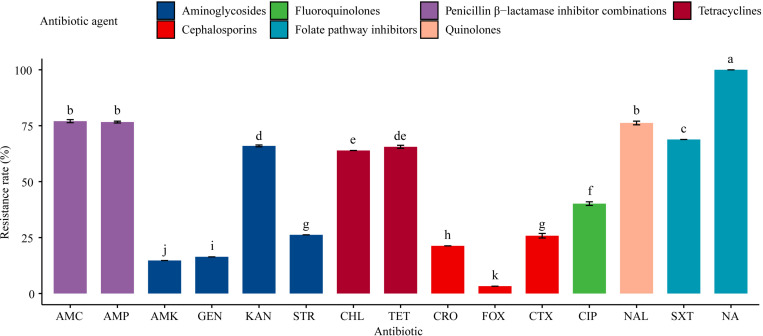
Resistance of 61 *Salmonella* isolates to 15 antibiotics. Table 1 has a list of abbreviations used in the abscissa. Different colors represent seven different antibiotic categories. Data are mean ± standard deviation (*n* = 3). Different letters used to label the bar chart denote significant differences found between the resistance rates of isolates to the corresponding antibiotic (*P* < 0.05).

All the 61 *Salmonella* isolates were resistant to sulfisoxazole (100%), and the rate of sulfisoxazole resistance was significantly (*P* < 0.05) higher than the rates of resistance to the other 13 antibiotics tested for (Figure 2). For example, 77.1% of the isolates were resistant to amoxicillin-clavulanate, ampicillin, and nalidixic acid, while nearly two-thirds (63.9–68.9%) of the tested isolates exhibited resistance to trimethoprim-sulfamethoxazole, kanamycin, tetracyclines, and chloramphenicol. Less than half (40.1%) of the isolates were resistant to ciprofloxacin; around one-fifth (21.3–26.2%) of the isolates were resistant to streptomycin, ceftiofur, and ceftriaxone; and the resistance rates to gentamicin, amikacin, and cefoxitin were relatively low (3.3–16.4%).

There were no significant differences in the resistance rates of *Salmonella* isolates to amoxicillin-clavulanate, ampicillin, and nalidixic acid; however, these resistance rates were significantly (*P* < 0.05) higher than the rates of resistance to the other antibiotics tested, except the resistance rate to sulfisoxazole. Additionally, significant (*P* < 0.05) differences were found in the resistance rates to amikacin, gentamicin, kanamycin, streptomycin, chloramphenicol, cefoxitin, ceftiofur, ceftriaxone, ciprofloxacin, and trimethoprim-sulfamethoxazole. Moreover, the resistance rates to aminoglycosides (amikacin, gentamicin, and kanamycin) and cephalosporins (cefoxitin, ceftriaxone, and ceftiofur) differed significantly (*P* < 0.05; Figure 2).

**FIGURE 3:**
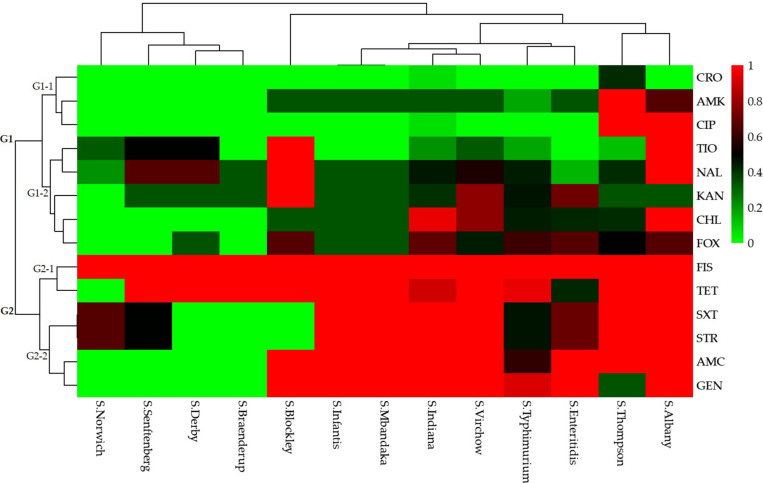
Heatmap (with dendrograms) showing the antibiotic susceptibilities of 61 *Salmonella* isolates among 13 serotypes. Red squares (pixels) represent high-frequency values and green squares represent low-frequency values. Table 1 presents a list of the abbreviations of the antibiotics.

A total of 49 (80.3%) *Salmonella* isolates were resistant to more than three categories of antibiotics. Specifically, 10 (20.4%) isolates were resistant to 3–5 categories (3–8 species) of antibiotics and the remaining 39 (79.6%) isolates were resistant to 6–8 categories (8–12 species) of antibiotics. The *Salmonella* isolates that were resistant to the 15 antibiotics based on different serotypes could be grouped into two clusters (G1 and G2; Figure 3).

Isolates resistant to ceftriaxone, amikacin, ciprofloxacin, ceftiofur, nalidixic acid, kanamycin, chloramphenicol, and cefoxitin were grouped in cluster G1. Of these, isolates resistant to ceftriaxone, amikacin, and ciprofloxacin were grouped in a secondary cluster (G1-1), and isolates resistant to the other five antibiotics were grouped in another secondary cluster (G1-2). Isolates resistant to sulfisoxazole, tetracyclines, trimethoprim-sulfamethoxazole, streptomycin, amoxicillin-clavulanate, and gentamicin were grouped in cluster G2. In this cluster, isolates resistant to sulfisoxazole and tetracycline were grouped in a secondary cluster (G2-1), and those resistant to trimethoprim-sulfamethoxazole, streptomycin, amoxicillin-clavulanate, and gentamicin were grouped in another secondary cluster (G2-2).

**FIGURE 4:**
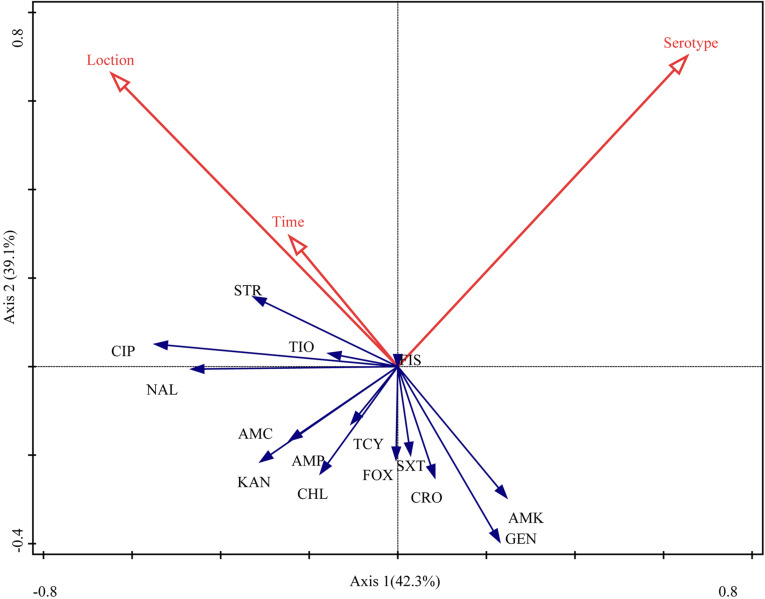
Relationship between the antibiotic susceptibility of *Salmonella* isolates, their serotype, sampling location, and sampling time. Table 1 presents a list of the abbreviations of the antibiotics. Blue arrow, species variable; red arrow, environmental variable. The cosine of the angle between two variables represents the correlation between them. For example, two variables that are nearly at a right angle to each other are almost uncorrelated.

The RDA biplot (Figure 4) indicated that the contribution of serotype to the antibiotic susceptibility of *Salmonella* isolates was the highest (42.3%, *P* = 0.004), followed by sampling location (39.1%, *P* = 0.018) and time (18.6%, *P* = 0.138). The serotype of the isolates was correlated with their susceptibility to most of the antibiotics tested for, except amoxicillin-clavulanate, ampicillin, kanamycin, and chloramphenicol. Additionally, sampling location and time were (closely) correlated with the susceptibility of the isolates to streptomycin, ciprofloxacin, ceftiofur, nalidixic acid, amoxicillin-clavulanate, ampicillin, kanamycin, tetracyclines, and chloramphenicol.

### PFGE Subtype of *Salmonella*

**FIGURE 5:**
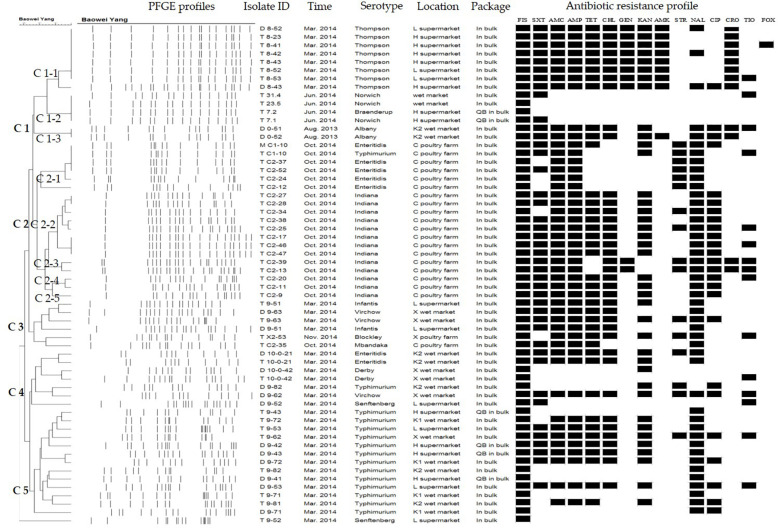
*Xba*I-based pulse-field gel electrophoresis (PFGE) and antibiotic resistance profiles of *Salmonella* isolates from eggs.

When the *Salmonella* isolates were subtyped by PFGE, the genomic DNA of each isolate produced 13–21 bands with the typing rate of 100% (Figure 5). Isolates derived from the eggs collected in the same month always belonged to the same serotype, and they exhibited the same or similar antibiotic resistance phenotype, despite being recovered from different locations (C1-1, C2-3, and C5). In contrast, some of the isolates recovered from the eggs sampled from different locations belonged to various serotypes, yet they also showed identical PFGE profiles and antibiotic resistance phenotypes (C1-2 and C1-3). Moreover, some isolates recovered from the eggs collected at the same location and time belonged to the same serotype, but they showed distinct PFGE and antibiotic resistance profiles (C2-2, C2-4, and C2-5).

## Discussion

The total egg production reached 24,446 million metric tons in 2013 and 4539 billion in 2014 (FAO, 2018a,b). If contaminated eggs were produced by laying hens infected with *Salmonella*, it would be difficult to effectively eliminate such contamination through vaccination and other interventions. Therefore, not only could this lead to outbreaks of foodborne illness in humans, but chickens hatched from contaminated eggs may also have inherent defects (Barrow, 2007; Kouam et al., 2018). The detection and characterization of *Salmonella* spp. in eggs can provide useful information for the mitigation of socioeconomic losses caused by contamination with *Salmonella*. In the present study, the average detection rate of *Salmonella*-positive eggs in poultry farms, supermarkets, and wet markets in Yangling (5.6%) was higher than those reported in Shandong Province (2.1%, 49/2342) and Hebei Province (4.8%) (Li et al., 2018; Yang et al., 2019); however, it was much lower than the detection rates in some conventional farms in Sichuan Province (12.2%) and Jiangsu Province (17.4%, 160/920), China (Li et al., 2013; Hai et al., 2020). During the study period, a high detection rate of *Salmonella*-positive eggs in 2014 was mainly found in the samples collected in March and October, and all the *Salmonella*-positive samples in October were obtained from the medium-scale poultry farm C. Although these results indicate that *Salmonella* is still prevalent in the eggs, the tendency of reduced prevalence suggests that in recent years, China has achieved remarkable results in monitoring and controlling the contamination of eggs with pathogens including *Salmonella* from farm to table.

Based on our results, the detection rate of *Salmonella*-positive eggs in the production (6.6%) and sales (5.1%) links were similar. A previous investigation provided a detailed description of contamination during five breeder farm production stages (i.e., laying, hatching, rearing, brooding, and post-hatching), with a considerably high prevalence of egg samples containing *Salmonella* found at the laying (29.2%) and hatching (21.6%) stages (Fei et al., 2017). In the current study, a total of 304 eggs were collected from three different poultry farms of different scales in Yangling. Although the number of eggs collected from each farm was similar, the detection rate of *Salmonella*-positive eggs varied in a broad range across different farms. Except one from the large-scale farm X, all the remaining *Salmonella-*positive eggs were detected from medium-scale farm C, while no *Salmonella*-positive eggs were detected from the small-scale farm F. Farm C was a conventional poultry farm with a smaller production scale and a higher density of breeding hens compared to poultry farm X. According to our observations during sampling, raw eggs in farm C all rolled to the same egg tray and were harvested by hand. Moreover, there was no separate space between the breeding and egg collection areas in farm C. Thus, cross-contaminations may be a major factor responsible for the high prevalence of *Salmonella* in the eggs collected from this farm. Conversely, in the large-scale and modernized farm X, the production units were completely closed off, with feed supply and egg collection occurring through different conveyor belts; this could reduce the chance of cross-contamination caused by *Salmonella* present in the environment, especially in the feces of hens. Changes in the management of stocking density at the laying stage can influence *Salmonella* contamination (Gast et al., 2014). In addition, immunological changes in breeder chickens at the laying stage can increase the contamination rates (Johnston et al., 2012).

*Salmonella* Typhimurium and *S.* Enteritidis are the two most commonly identified serotypes and causative agents involved in foodborne salmonellosis (Galis et al., 2013; Whiley and Ross, 2015). In the present study, 13 different serotypes were identified from the 61 *Salmonella* isolates from the eggs. The top four serotypes were *S.* Typhimurium, *S.* Indiana, *S.* Thompson, and *S.* Enteritidis. Generally, our results were consistent with the results of previous studies reporting that *S.* Typhimurium was the most prevalent serotype in eggs obtained from Penang in Malaysia and 50 poultry farms throughout Korea (Adzitey et al., 2012; Bae et al., 2013). Additionally, *S.* Indiana, *S.* Typhimurium, and *S.* Enteritidis were found to be the predominant *Salmonella* serovars in eggs sampled in Yangzhou, China (Li et al., 2017), while *S.* Enteritidis was the most prevalent serotype in eggs collected from Shandong, Shanghai, and Guangdong in China (Ni et al., 2018; Xie et al., 2019; Yang et al., 2019). Particularly, *S.* Enteritidis has always been a common foodborne pathogen associated with salmonellosis caused by consumption of contaminated eggs or egg products, and this is mainly due to its specific survival mechanism in egg white with the assistance of the outer membrane channel TolC (Huang et al., 2019; Raspoet et al., 2019). Here, although *S.* Enteritidis was not the most dominant serotype in all egg samples, over 70% of *S.* Enteritidis isolates were derived from poultry farms, and the remaining isolates were recovered from wet markets. It could be considered that *S.* Enteritidis remained to be the predominant serotype of *Salmonella* in contaminated eggs from poultry farms in the study area.

In total, 10 *Salmonella* serotypes were detected in the isolates derived from retail eggs, while five serotypes were identified from eggs collected from poultry farms. Both *S.* Typhimurium and *S.* Enteritidis were detected in the eggs from poultry farms and retail markets, whereas the other eight serotypes were detected in the eggs from retail markets only. Interestingly, *S.* Indiana and *S.* Blockley isolates that were commonly detected from poultry farms were absent in the eggs from marketplaces. These results indicate that the prevalent serotypes of *Salmonella* in eggs differed between retail markets and poultry farms. Previous studies have reported that the pathways of pathogen contamination could be influenced by the egg production, storage, and handling procedures; in other words, cross-contamination may occur during egg storage, transportation, and sales, although some *Salmonella* strains die in these periods (Namata et al., 2008; Food Drug Administration, 2009; Gast et al., 2014). In addition, *S.* Indiana accounted for a large proportion (23.0%; 15/61) of the *Salmonella* isolates from eggs in the current study, and it was frequently detected in poultry in China during 2006–2012 (Gong et al., 2017). However, this serotype has not been commonly documented in other countries. Since *Salmonella*, especially *S.* Typhimurium, could maintain a high survival rate on eggshells and *S.* Enteritidis within the egg, potential multiple outbreaks associated with these two serotypes in eggs could occur (McAuley et al., 2015). Therefore, apart from improving the hygienic conditions of egg production, efficient measures should be implemented to reduce exposure and surface contact of eggs during transportation and handling in order to prevent contamination by foodborne pathogens including *Salmonella*.

In poultry rearing, excessive antibiotics are commonly used for disease prevention and growth promotion, leading to the occurrence of antibiotic-resistant bacteria (Krishnasamy et al., 2015; Haskell et al., 2018). A considerable increase in multidrug-resistant (MDR) *Salmonella* has been observed, and the number of MDR *Salmonella* infections has increased worldwide over the past few years (Fardsanei et al., 2018). In the present study, all the 61 *Salmonella* isolates from eggs exhibited resistance to one or more antibiotic agents, and only three isolates were non-MDR. Notably, one-third of the isolates were resistant to at least 10 antibiotics. Similarly, Al et al. (2015) found that the rate of MDR *Salmonella* isolated from eggs and poultry products reached up to 86.5%, and *S.* Thompson isolates exhibited higher antibiotic resistance than isolates of other serotypes recovered from the same marketplace. Moreover, Sanchez-Salazar et al. (2019) obtained 31 *Salmonella* isolates from layer poultry farms in central Ecuador in 2017, 58.1% (18/31) of which were MDR. Taken together, these findings corroborate reports showing that MDR *Salmonella* is prevalent in eggs (Garedew et al., 2015; Bezerra et al., 2016; Taddese et al., 2019). In the current study, almost all *S.* Indiana and *S.* Enteritidis isolates derived from poultry farm C were MDR strains. This result might be attributed to excessive use and overdosage of antibiotics in poultry farm C associated with poor production environment and sanitary conditions.

According to the PFGE profiles, some *Salmonella* isolates within the same serotype were derived from the same location and/or time; these isolates had a close genetic relatedness while they shared the same or similar antibiotic resistance phenotypes. This result is consistent with a previous study of *Salmonella* contamination in layer poultry farms in Shandong and Hebei Provinces, China, that certain samples within henhouses and egg-collecting areas showed relatively high genetic similarities (Li et al., 2018). Here, almost all the *Salmonella* isolates recovered from poultry farms were identified to be the same serotype with similar PFGE and antibiotic resistance profiles. Likewise, the *Salmonella* isolated from different backyard eggs in Portugal displayed identical PFGE profiles, indicating that they belonged to a prevalent clone in the region (Ferreiraa et al., 2020). Moreover, some isolates of a certain serotype sampled from different locations and at different times exhibited the same or similar PFGE profiles and antibiotic resistance phenotypes. This result indicates that *Salmonella* isolates of these serotypes might have existed in the parturient canal of laying hens, poultry rearing environments, or transportation and storage systems for a long period of time. In contrast, some isolates of the same serotype showed diverse PFGE profiles and antibiotic resistance phenotypes. From a poultry slaughterhouse in Brazil, 40 *Salmonella* isolates obtained over a 20-week period showed diverse PFGE profiles in the same serotype, except *S.* Enteritidis, which could occur due to the low genetic diversity in this serovar (Dantas et al., 2020). Our results indicate that the prevalence of *Salmonella* in eggs could pose potential hazards for consumers and result in *Salmonella* outbreaks over a certain period of time.

This study indicated that *Salmonella* was prevalent in fresh eggs from poultry farms and retail marketplaces with diverse serotypes, and the majority of the isolates were resistant to the multiple antibiotics tested. Some isolates of the same serotype were sampled from the same location and/or time, which shared identical or highly similar PFGE profiles and antibiotic resistance profiles. As eggs play a vital role in daily human life and can be easily contaminated by MDR *Salmonella*, it is crucial for local health departments to monitor the occurrence of *Salmonella* in eggs and prevent egg contamination via the food production and supply chains, including poultry farms and retail markets.

## Data Availability Statement

All datasets generated for this study are included in the article/supplementary material.

## Author Contributions

WL conceived the study and drafted the manuscript. WL, HL, SZ, ZW, and QN performed the experiments and collected the data. HS conducted data analysis. BY supervised the project, experimental design, data collection and analysis, and manuscript preparation. CS and XS guided the experimental design and assisted with manuscript revision. All authors contributed to manuscript revision and agreed to the published version of the manuscript.

## Conflict of Interest

The authors declare that the research was conducted in the absence of any commercial or financial relationships that could be construed as a potential conflict of interest.

## Footnotes

1 https://rstudio.com/

2 https://www.rdocumentation.org/packages/pheatmap/versions/1.0.12
